# A Fatal Case of Necrotizing Soft Tissue Infection Caused by Aeromonas hydrophila Starting in the Thigh

**DOI:** 10.7759/cureus.78446

**Published:** 2025-02-03

**Authors:** Masayuki Yonezu, Toshiro Imamoto

**Affiliations:** 1 Department of Emergency Medicine and Critical Care, Saitama Medical Center, Saitama Medical University, Kawagoe, JPN

**Keywords:** aeromonas hydrophila, hip disarticulation, immunocompromised host, nsti, thigh

## Abstract

Necrotizing soft tissue infection (NSTI) remains a highly fatal disease. Among its causes, *Aeromonas hydrophila* is a Gram-negative bacillus endemic in freshwater environments that can cause fatal systemic infections in compromised hosts. The most important treatment of NSTI is source control done as soon as possible.

In this report, we describe a case of right femoral NSTI caused by *Aeromonas hydrophila* that was diagnosed based on minor physical findings, in which the patient died, despite early hip dissection. An 82-year-old man was admitted because of right femoral NSTI. Thirty hours after admission, we performed a right hip disarticulation for source control, and *Aeromonas hydrophila* was detected in the wound culture on the same day. Despite continuous treatment, he developed fungemia due to candida, eventually leading to his death.

In general, infection in a typical NSTI spreads from the extremities to the trunk, and appropriate debridement can save the patient's life. In the present case, the infection started from the thigh and may have progressed more rapidly than in other cases of NSTI.

It is important to assume *Aeromonas hydrophila* as the causative organism of NSTI in a compromised host, regardless of the history of exposure to a freshwater environment, depending on the patient's background and Gram staining results. Even if physical findings appear mild, a decision to perform hip dissection or pelvic hemisection for quick and aggressive source control may save the lives of similar patients.

## Introduction

The median fatality rate of necrotizing soft tissue infection (NSTI) remains high, at 32.2%, and its prevalence globally has been reported to be 0.40 cases per 100,000 population. The disease affects all age groups, although middle-aged and elderly patients (over 50 years of age) are more likely to be infected. The most common risk factor for the development of NSTI is diabetes mellitus. The other comorbidities include obesity, hypertension, alcohol abuse, liver cirrhosis, chronic renal failure, immunodeficiency, and peripheral vascular disease. Infection begins in the hypodermis or superficial fascia. Invasive bacteria cause thrombosis of the nutrient vessels in the subcutis. Necrosis of hypodermis and superficial fascia is directly related to bacterial enzymes that destroy fascia and fat and is secondarily of vascular origin. Tissue ischemia promotes infectious dissemination, later leading to skin necrosis and, when nerve branches are involved, severe pain. Gas formed by anaerobic bacteria may cause crepitus [1]. In the treatment of NTSI, it is essential to perform debridement as early and reliably as possible [2]. Wong et al. retrospectively reviewed the medical records of 89 consecutive patients who had been admitted to their institution for NSTI from January 1997 to August 2002. It reported that the mortality rate of patients treated surgically more than 24 hours after the initial diagnosis is 9.4 times higher than that of patients treated within 24 hours, and the presence or absence of surgical treatment is directly related to saving lives [3].

*Aeromonas hydrophila* is a facultative anaerobic Gram-negative rod that is endemic in freshwater environments such as sewage and wastewater and soil. It is non-spore-forming with rounded ends that measure 1-3.5 μm across. It can thrive at temperatures ranging from 0℃ to 45℃ with an optimum temperature of 22°C-32°C [4,5]. It can cause systemic infections such as NSTI in compromised hosts and requires prompt treatment because of its fatal course.

In this report, we describe a case of right femoral NSTI caused by* Aeromonas hydrophila* that was diagnosed from minor physical findings and treated with hip dissection at an early stage but resulted in the patient's death after a dramatic course. This paper does not require approval by an ethics committee as it is a case report. The patient's family has given their consent for publication.

## Case presentation

The patient was an 82-year-old man with a history of castration-resistant prostate cancer with liver metastasis (stage IV, clinical TNM classification: tumor, 4; nodes, 1; metastasis, 1). The patient had been treated with docetaxel for 10 months about two years prior to admission. After local radiotherapy and antihormonal therapy, he had been treated with cabazitaxel therapy for about six months prior to admission, and the disease onset was during the seventh course of cabazitaxel therapy. He had been taking prednisolone 10 mg per day as an oral anticancer drug combination since the beginning of the chemotherapy period.

The patient had been feeling unwell and aware of right lower extremity pain since the day before admission to the hospital, but he had not sought medical assistance. About 24 hours later, on the morning of the day of admission, he fell into a coma, and an emergency medical service was called. According to the patient and his family, there was no history of exposure to a freshwater environment.

**Figure 1:**
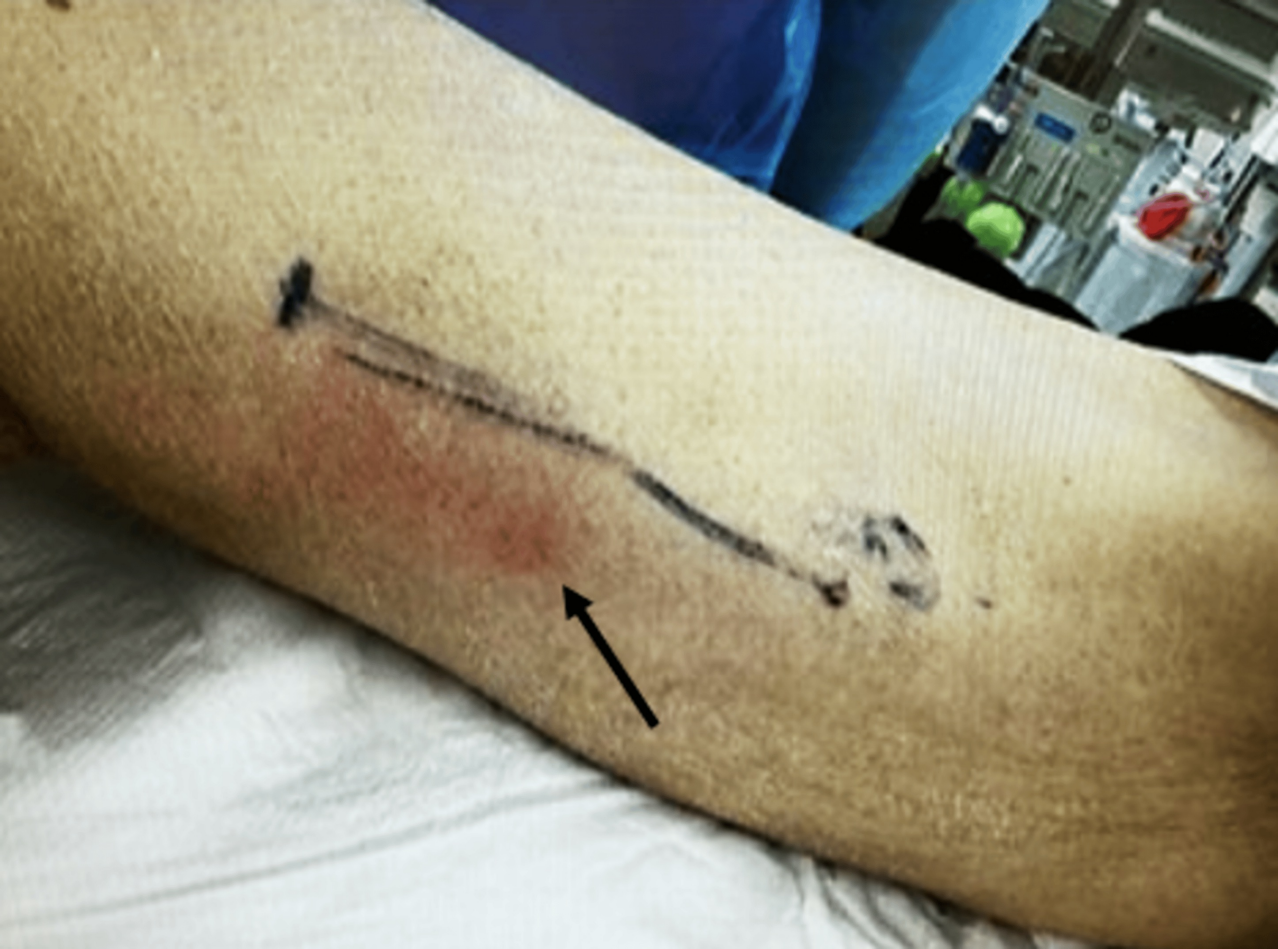
Initial skin inspection at initial presentation There is mild erythema of 10 cm diameter long on the distal skin of the right medial thigh (black arrow).

The initial vital signs were as follows: Glasgow Coma Scale, 15 (eye-opening: 4, best verbal response: 5, best motor response: 6); respiratory rate, 24 beats/minute; saturation of percutaneous oxygen, unmeasurable; pulse rate, 158 beats/minute; systolic blood pressure, 69 mmHg; diastolic pressure, unmeasurable; and body temperature, 39°C. The patient was in a marked state of shock. Physical examination revealed mild erythema of 10 cm diameter long on the distal skin of the right medial thigh, but no other findings such as swelling and crepitus (Figure 1).

The pain was severe and deviated from the impression of skin findings. Blood tests on admission showed marked myelosuppression, elevated CRP, acute kidney injury (AKI) (KDIGO stage 1), and elevated creatine kinase (CK) and transaminases (Table 1).

**Table 1:** Blood tests on day 1 and day 24 The results indicate marked myelosuppression. CPK: creatine phosphokinase

Parameter	Reference range	Day 1	Day 24	Unit of measure
Hematology				
White blood cell	3.3-8.6 × 10^3^	0.9 × 10^3^	0.7 × 10^3^	/μL
Neutrophils	42.4-75.0	36.8	57.8	%
Lymphocytes	18.2-47.7	50	19.7	%
Monocytes	3.3-9.0	12	19.7	%
Hematocrit	40.7-50.1	32.5	25.4	%
Hemoglobin	13.7-16.8	10.7	8.6	g/dL
Red blood cell	4.35-5.55 × 10^4^	332 × 10^4^	291 × 10^4^	/μL
Mean corpuscular volume	83.6-98.2	95.4	87.3	fL
Platelet count	15.8-34.8 × 10^4^	4.2 × 10^4^	1.5 × 10^4^	/μL
Activated partial thromboplastin time	24.1-31.7	20	46.5	Second
Prothrombin time %	74.4-120.0	88.3	51.1	%
Blood chemistry				
Total bilirubin	0.4-1.5	2.2	22.9	mg/dL
Direct bilirubin	0.0-0.2	1.3	18.9	mg/dL
Aspartate transaminase	13-30	62	247	U/L
Alanine transaminase	10-42	105	115	U/L
Total protein	6.6-8.1	5.2	3.5	g/dL
Serum albumin	4.1-5.1	3	1.6	g/dL
Sodium	138-145	137	136	mEq/L
Potassium	3.6-4.8	3.5	4.7	mEq/L
Chloride	101-108	100	106	mEq/L
Urea nitrogen	8-20	26	28	mg/dL
Creatinine	0.65-1.07	1.72	1.15	mg/dL
Glucose	73-109	93	210	mg/dL
CPK	59-248	103	42	U/L
C-reactive protein	0.00-0.14	11.34	12.15	mg/dL

**Figure 2:**
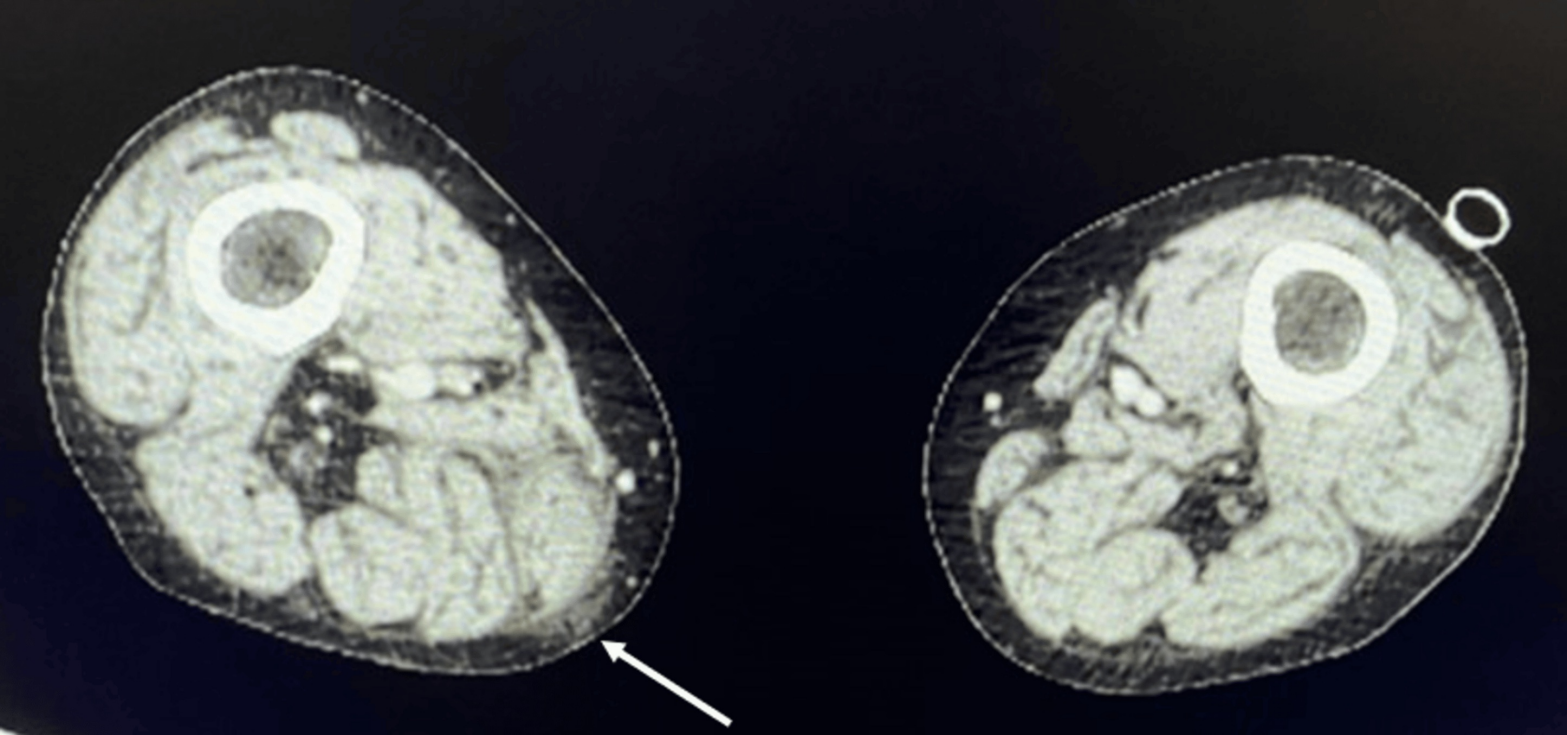
Contrast-enhanced CT at initial presentation There is edema around the gracilis muscle on the medial side of the right thigh (white arrow). CT: computed tomography

Contrast-enhanced computed tomography (CT) showed edematous changes around the gracilis muscle on the medial side of the right thigh, but no other findings (Figure 2).

**Figure 3:**
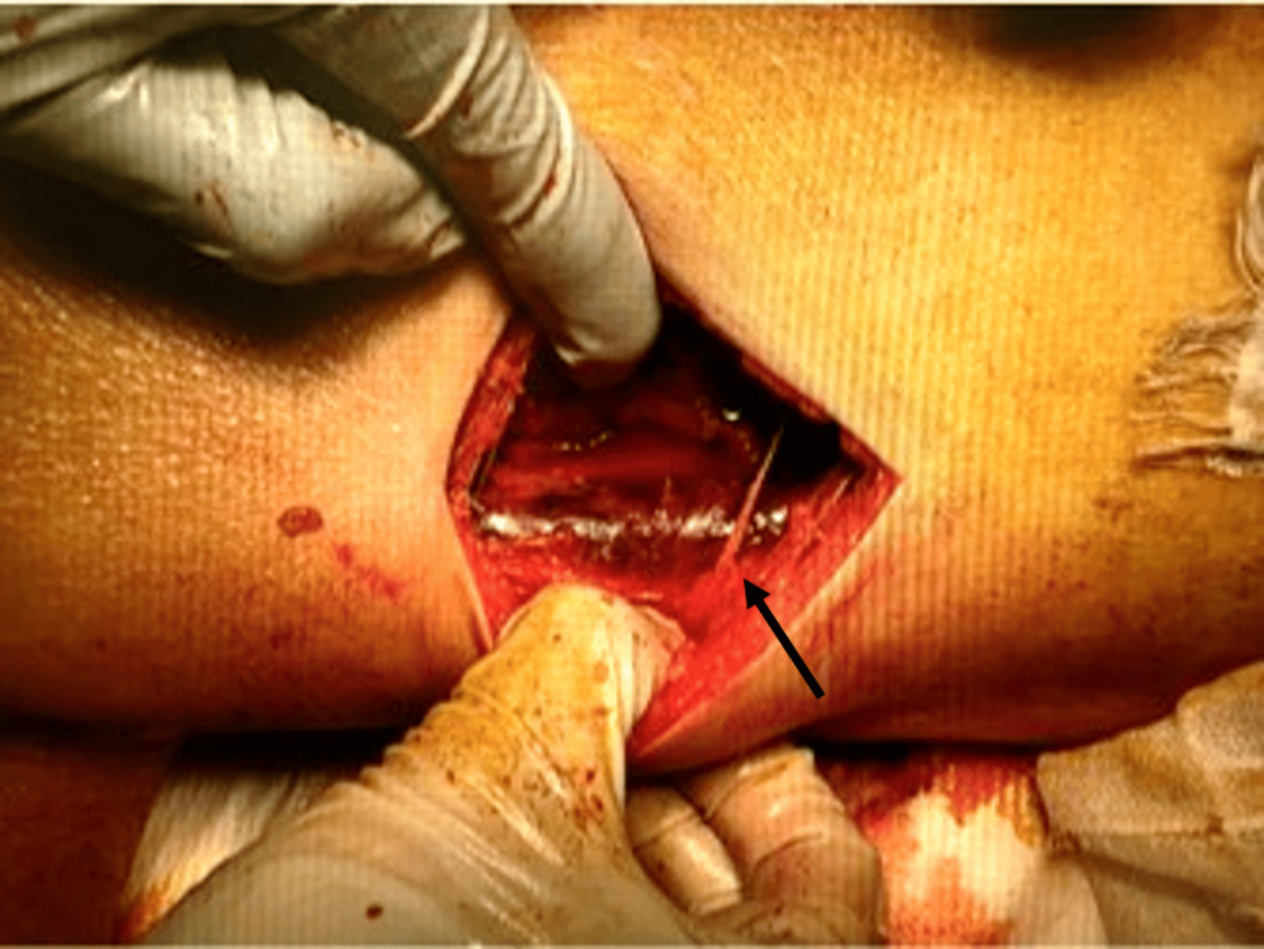
Findings at the time of the test incision Incising down to the deep fascia and probing of the index finger, dark exudate is seen along with a dark red myoid body of the gracilis muscle, suspicious for insufficiency of blood flow (black arrow). There was no bleeding associated with the incision.

However, based on the patient's marked shock and the results of the physical examination, blood tests, and CT, the decision was made to treat the patient diagnostically as having NSTI of the right thigh. A test incision made in the erythematous area of the right thigh revealed the presence of turbid exudate and a dark red myoid body that was suspicious for insufficiency of blood flow (Figure 3).

Gram staining of the exudate identified Gram-negative bacillus. The rapid group A streptococcal antigen test was negative.

**Figure 4:**
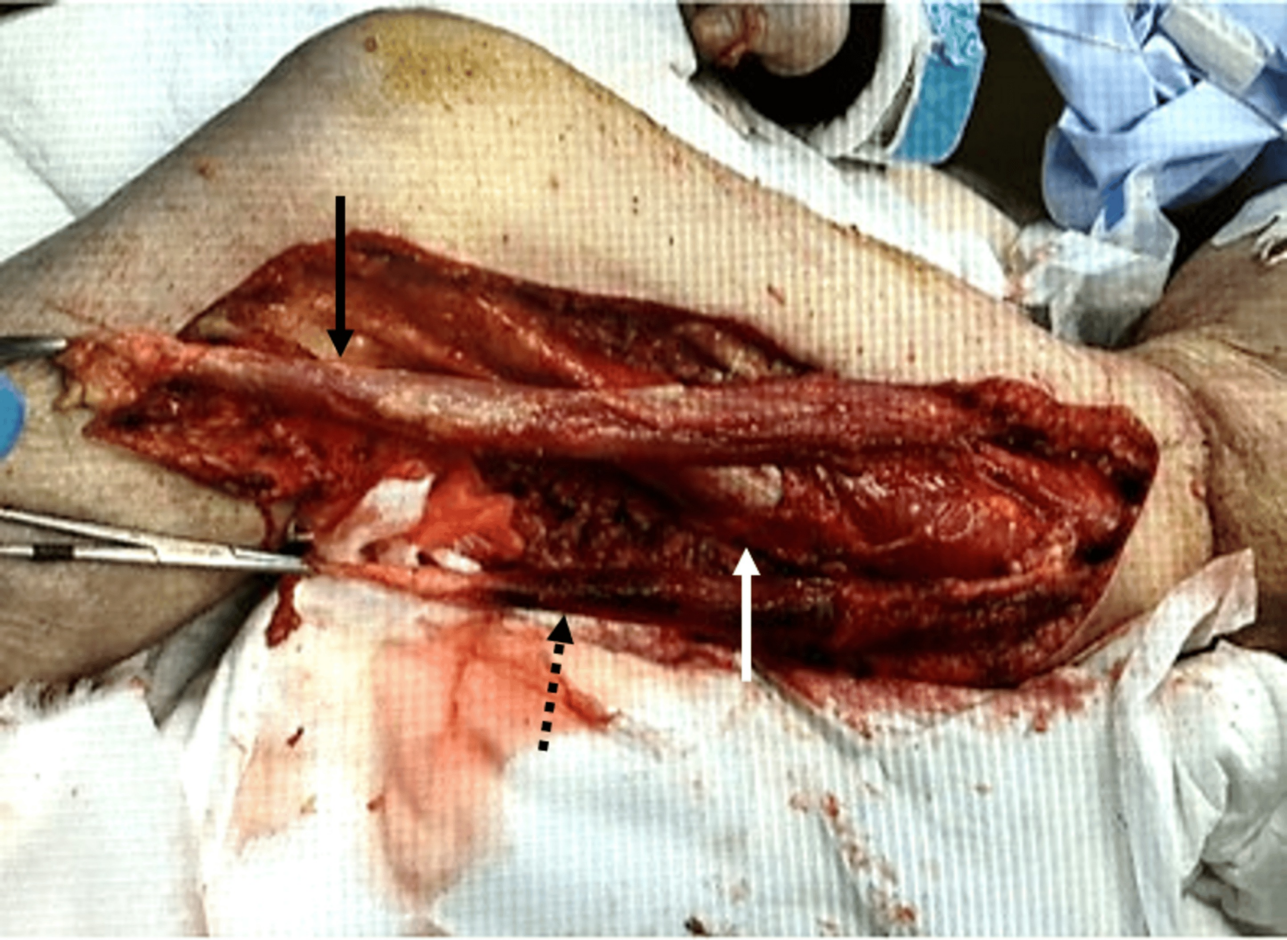
Intraoperative photograph during debridement After admission, the thigh was debrided, and the sartorius muscle (black arrow), gracilis muscle (black dotted arrow), and vastus medialis muscle (white arrow) were resected.

Based on the results of the test incision and Gram staining, we diagnosed septic shock due to right femoral NSTI caused by a Gram-negative bacillus. For septic shock, 3 liters of extracellular fluid was administered via a peripheral venous line according to the Surviving Sepsis Campaign Guidelines. For local treatment, after cleaning the wound and submitting wound culture and blood culture, treatment was started immediately with meropenem (MEPM) 1 g/day, vancomycin (VCM) 1.5 g/day, and clindamycin (CLDM) 2700 mg/day. A central venous catheter was inserted through the right internal jugular vein, and noradrenaline and vasopressin were administered. Hydrocortisone 200 mg/day was also started for critical illness-related corticosteroid insufficiency. The patient required intubation and ventilatory management for marked shock. After admission to the intensive care unit, the right thigh was debrided, and the sartorius, gracilis, and vastus medialis muscles were resected, but the lactic acidosis rapidly progressed, and purpura appeared on the thigh (Figure 4).

**Figure 5:**
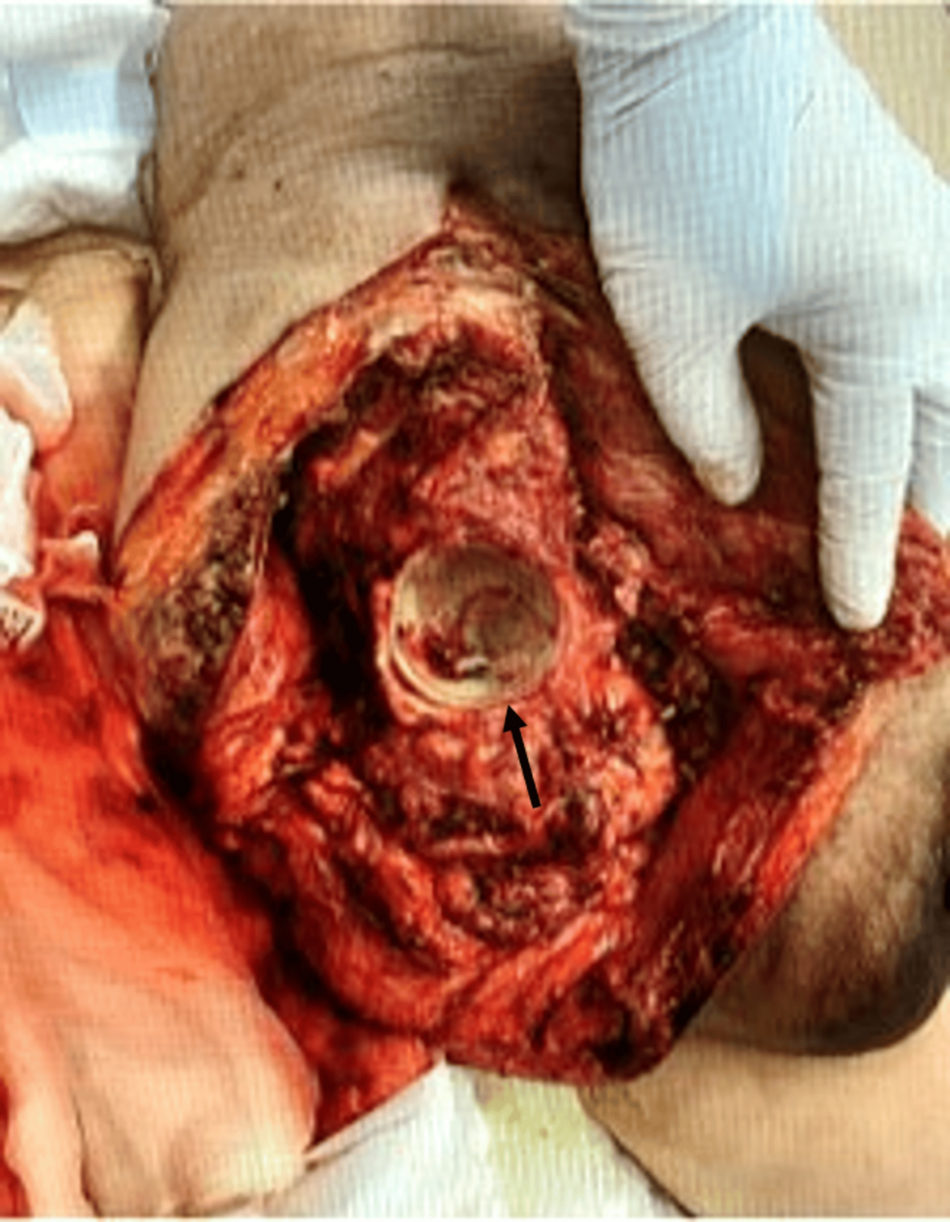
Intraoperative photograph at the time of hip disarticulation Right hip disarticulation was performed 30 hours after admission. There is an acetabulum (black arrow).

**Figure 6:**
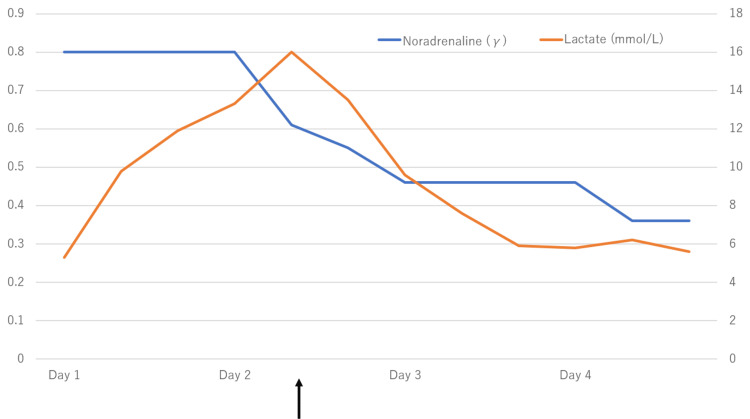
Noradrenaline dosage and lactate: right hip disarticulation course from day 1 to day 4 The lactic acidosis rapidly progressed, and purpura appeared on the thigh. Thirty hours after admission, right hip disarticulation was performed for source control (black arrow).

For source control, right hip disarticulation was performed 30 hours after admission (Figures 5, 6).

**Figure 7:**
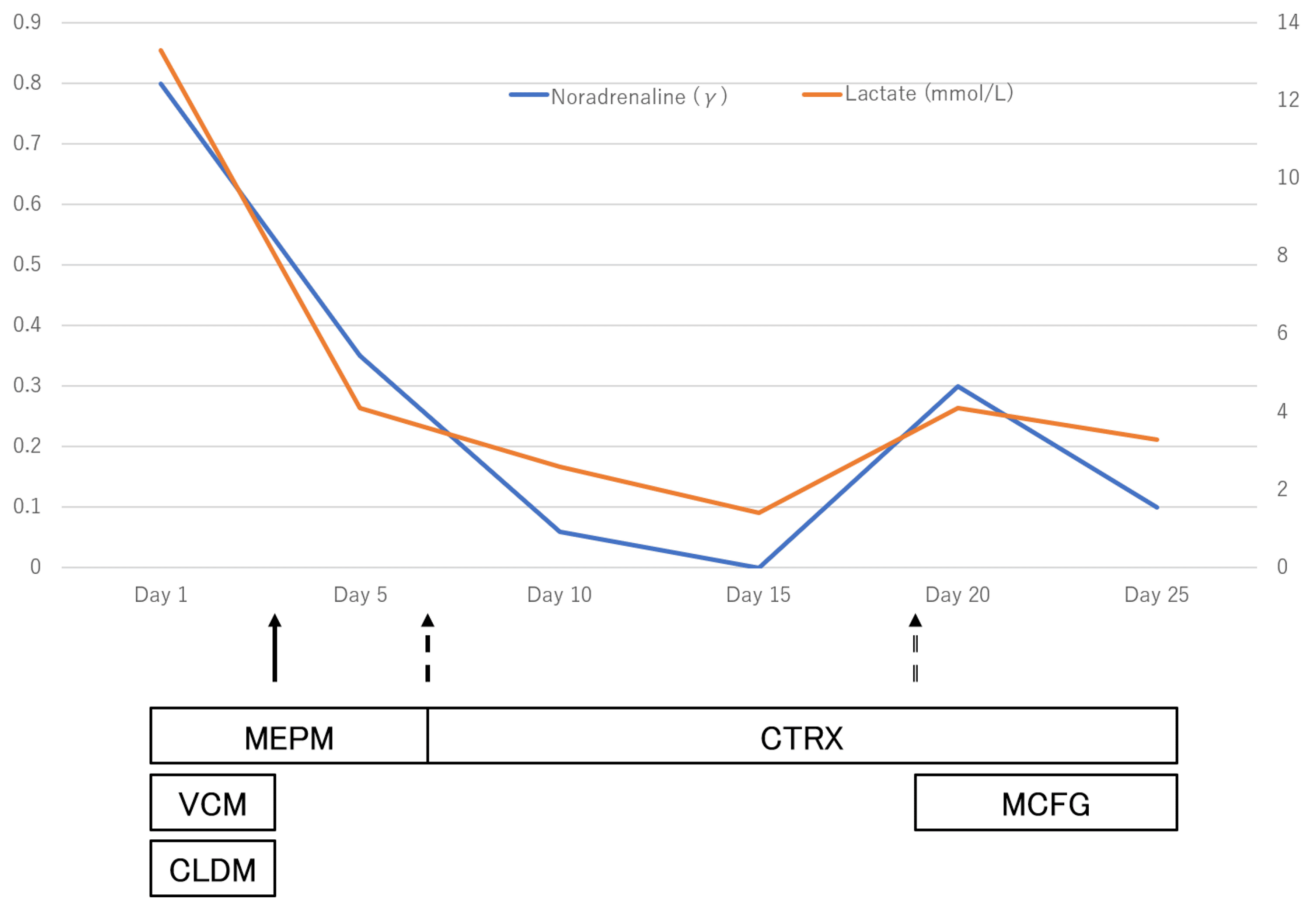
Noradrenaline dosage and lactate: antimicrobial course On the third day,* Aeromonas hydrophila *was detected in the wound culture (black arrow). On the seventh day, the sensitivity of *Aeromonas hydrophila* was found (black dotted arrow). On the 19th day, *Candida parapsilosis* was detected in the blood culture (black double double arrow). On the 17th day, the demand for vasopressors increased once more. MEPM: meropenem, VCM: vancomycin, CLDM: clindamycin, MCFG: micafungin, CTRX: ceftriaxone

Although the lactic acidosis improved, multiple organ failure was observed, including AKI, coagulopathy, and thrombocytopenia, requiring high doses of vasopressors. On the same day, *Aeromonas hydrophila* was detected in the wound culture on admission, and VCM and CLDM were terminated for de-escalation of antimicrobial agents. On the fourth day after admission, renal replacement therapy was started for metabolic acidosis. On the seventh day, sensitivity of *Aeromonas hydrophila* was found, and the antimicrobial agent was de-escalated from MEPM to ceftriaxone 2 g/day. The patient's condition began to improve. On the 15th day, the patient was weaned from ventilator management, and the demand for vasopressors was eliminated. However, progression of soft tissue necrosis in the wound was observed, the patient's blood pressure dropped on the 17th day, and the demand for vasopressors increased once more (Figure 7).

On the 19th day, *Candida parapsilosis* was detected in wound culture, and the blood culture was submitted. On the 21st day, *Candida parapsilosis* was detected in the blood culture. Micafungin 100 mg/day was started as a treatment for deep mycosis, but *Candida parapsilosis* continued to be detected in subsequent retests of blood and wound cultures. On the 24th day, blood test results were suggestive of significant myelosuppression (Table 1). We judged that it would be difficult to save the patient's life, and the family was informed of the patient's condition on the 25th day. With their consent, we made the decision to withhold further treatment. Despite continuous multidisciplinary treatment from the day of admission, the patient died on the 26th day.

## Discussion

*Aeromonas hydrophila* is a facultative anaerobic Gram-negative bacillus that is endemic in freshwater environments such as sewage and wastewater and soil [4,5]. It is a common cause of diarrhea, wound infection, and bacteremia [6] but can also cause soft tissue infections and meningitis. Because of its low pathogenicity, it is usually a problem when it is a causative agent of opportunistic infections. In compromised hosts, systemic infections such as NSTI may occur that require prompt treatment because of the fatal course of the disease [7,8]. The mortality rate of NSTI caused by *Aeromonas hydrophila* is 80%, and it has been reported that the mortality rate increases to almost 100% in the presence of bacteremia or multiple organ failure complicating NSTI that is caused by this organism [9]. In the present case, *Aeromonas hydrophila* was detected in the wound culture, and multiple organ failure was observed, including AKI, coagulopathy, and thrombocytopenia, indicating a high likelihood of mortality.

The initial skin features of NSTI are erythema and calor. As the infection progresses, blistering, purpura, and dysesthesia are seen [10], of which blistering was a helpful clinical feature in the early diagnosis [11]. The most important treatment for NSTI is source control done as soon as possible. Specifically, it is recommended that within 24 hours of the initial debridement, a re-evaluation and debridement of the wound should be continued daily until all necrotic tissue has been removed and only healthy tissue remains [10,12,13]. It has also been reported that performing the first fasciotomy and radical debridement within 24 hours of symptom onset significantly improves survival [2,14], suggesting the need for a more rapid response in the present patient.

The above indicates that survival is remarkably low in compromised hosts with NSTI caused by *Aeromonas hydrophila* and that debridement is essential. Most reported cases of NSTI caused by *Aeromonas hydrophila* have resulted in death [15-22]. However, another report describes a case that was saved by appropriate debridement [9]. Our case was undergoing chemotherapy for carcinoma and was a long-term steroid user. He had bone marrow suppression that may have been caused by chemotherapy or severe sepsis on blood test at the time of transport, so it was known immediately after treatment that the patient was a compromised host.

The course of the present case is characterized by the fact that although there was no history of exposure to a freshwater environment and the initial physical examination was relatively unremarkable, with only erythema of the skin, the treatment and hip disarticulation performed 30 hours after admission was not effective, and the patient developed multiple organ failure. Although the hip joint was dissected at a very early stage, the infection was still not under control, leading to multiple organ failure and deep mycosis.

A typical NSTI starts at the distal end of an extremity and spreads to the trunk. In the present case, the infection originated in the thigh, and it is possible that the infection progressed to the trunk more rapidly than in typical cases. Infection typically spreads from the toes and other parts of the body, and below-knee amputation or above-knee amputation (AKA) with appropriate debridement can be performed to save the patient's life, as has been reported in many cases [23-28]. More extensive debridement than AKA includes hip dissection and pelvic hemiarthroplasty. As these are extremely invasive for the patient, it is not easy for the emergency physician or orthopedic surgeon in charge to decide on these procedures immediately after diagnosis [9,29-31].

It is important to assume *Aeromonas hydrophila* as the causative organism in NSTI of compromised hosts, regardless of whether the patient has a history of exposure to a freshwater environment, depending on the patient background and the results of Gram staining [32]. Even if the physical findings are minor, the decision to perform a hip dissection or pelvic hemisection can be made more confidently based on the results of Gram staining, sometimes simultaneously with diagnosis. More rapid source control along with sufficient debridement may save the lives of those such as the present patient, who could not be saved this time.

## Conclusions

NSTI caused by *Aeromonas hydrophila *in a compromised host has a significantly poor prognosis. It is important to estimate the causative organism by Gram staining to make an early diagnosis. Even if the physical findings are slight, it may be necessary to be willing to perform debridement earlier and more extensively, depending on the site of onset of the disease.
